# Are testicular sperms superior to ejaculated sperms in couples with previous ART failure due to high rate of fragmented embryos? A retrospective cohort study

**DOI:** 10.3389/fsurg.2022.1065751

**Published:** 2023-01-06

**Authors:** Ling-Ying Jiang, Fei-Fei Kong, Lv Yao, Fu-Xing Zhang, Sha-Sha Wang, Xiao-Ying Jin, Xiao-Mei Tong, Song-Ying Zhang

**Affiliations:** ^1^ Assisted Reproduction Unit, Department of Obstetrics and Gynecology, Sir Run Run Shaw Hospital, Zhejiang University School of Medicine, Hangzhou, China; ^2^ Key Laboratory of Reproductive Dysfunction Management of Zhejiang Province, Hangzhou, China

**Keywords:** assisted reproductive therapy (ART), intracytoplasmic sperm injection (ICSI), ejaculated sperm, testicular sperm aspiration (TESA), embryo fragmentation, cycle outcome

## Abstract

**Objective:**

The aim was to clarify whether using testicular sperm reduces embryo fragmentation and improves cycle outcomes.

**Methods:**

Fragmented embryo was defined as an embryo in which fragments account for more than one third of the embryonic surface area. High rate of fragmented embryos was defined by a proportion of fragmented embryos higher than 50%. We recruited infertile couples who had undergone at least one ovarian stimulation cycle using ejaculated sperm but failed to conceive due to high rate of fragmented embryos in each previous cycle. After fully informed consent, the couples agreed to obtain testicular sperm by testicular puncture and use testicular sperm for intracytoplasmic sperm injection (ICSI). The normal fertilization rate, transferable embryo rate, fragmented embryo rate and cycle outcomes were compared between ejaculated sperm group (EJA-sperm group) and testicular sperm group (TESTI-sperm group).

**Results:**

Twenty-two couples who agreed to participate in our study underwent 32 ICSI cycles with ejaculated spermatozoa and 23 ICSI cycles with testicular spermatozoa. Embryo transfers were cancelled in 8 ejaculated cycles and 4 testicular cycles because of no transferable embryos. There were no significant differences in age, normal fertilization rate and high-quality embryo rate between ejaculated and testicular groups. The transferable embryo rate and implantation rate in TESTI-sperm group were significantly higher than those in EJA-sperm group (36.9% vs. 22.0%, *p* < 0.01; 34.2% vs. 0%, *p* < 0.001). The fragmented embryo rate in TESTI-sperm group was significantly lower than that in EJA-sperm group (61.2% vs. 75.7%, *p* < 0.05).

**Conclusion:**

Our small retrospective cohort study suggests that using testicular sperm may be a recommended option for couples with previous ART failure because of high rate of fragmented embryos. Large samples, multicenter studies or randomized controlled trial (RCT) are needed to further confirm the superiority of testicular sperm.

## Introduction

Nowadays, assisted reproductive therapy (ART) is widely used in the treatment of infertility (1, 2). In particular, ICSI technology solves the fertilization problem of severe oligozoospermia or severe asthenozoospermia (3).

Patients with obstructive azoospermia usually undergo a vasovasostomy or vasoepididymostomy to attempt to reopen the vas deferens or epididymis. However, if no sperm is found in the semen after surgery, ICSI with testicular sperm obtained by puncture or biopsy is also an effective way to treat infertility (4).

Compared with ejaculated sperm, testicular sperm bypasses genital tract, long abstinence, and gradient centrifugation process and thus is speculated of having better quality (5–9). Because of this, researchers have tried using testicular sperm replacing ejaculated sperm to treat other types of infertility (5, 10–14). Weissman et al. (2008) reported that four couples with severe oligoteratoasthenozoospermia (OTA) who failed to achieve successful pregnancies after recurrent induction of ovulation had all achieved ongoing pregnancies after using testicular sperm (10). Some investigators compared the outcome of ovulation induction cycles using either ejaculated or testicular sperm in men diagnosed with cryptozoospermia, showing a trend favoring testicular sperm (11–13). Esteves et al. (2018) and Zhang et al. (2019) suggested that using testicular sperm is an effective option to overcome infertility when applied to selected men with high sperm DNA fragmentation index (DFI) (5, 14).

Fragmentation, or anucleate cell fragments generated during early embryo development, has been shown to be an important biomarker for embryo quality and implantation potential (15). Thus, assessment of fragmentation is an important part of embryo scoring system. Highly fragmented embryos have poor ability to form blastocysts, causing patients to have little chance of embryo transfer (16). Recurrent development of fragmented embryo represents one of the greatest challenges for gynecologists and embryologists. There are many reasons for embryo fragmentation, but in fact, the exact cause is not clear (17). Considering that testicular sperm is speculated of having better quality than ejaculated sperm and can be used for patients with OTA or high sperm DFI, we tried to use testicular sperm for patients with previous ART failure because of high rate of fragmented embryos.

Based on the Istanbul embryo scoring consensus (18), we defined fragmented embryo and high rate of fragmented embryos. We recruited infertile couples who had failed to conceive in previous ejaculated stimulation cycles according to the inclusion and exclusion criteria that would be mentioned in the methods section. Thirty-seven couples fulfilled the inclusion criteria, 22 of which agreed to participate in this study and accepted to use testicular sperm in later stimulation cycles. The aim of this study was to clarify whether using testicular sperm reduces embryo fragmentation and improves cycle outcomes.

## Materials and methods

### Patient selection

A single-center retrospective cohort study was conducted between January 2019 and December 2021 in Assisted Reproduction Unit of Sir Run Run Shaw Hospital, Zhejiang University School of Medicine, Hangzhou, China. Fragmented embryo was defined as an embryo in which fragments account for more than one third of the embryonic surface area. High rate of fragmented embryos was defined by a proportion of fragmented embryos higher than 50%. The following inclusion criteria were established: (1) infertility duration >1 year; (2) women ages 38 years old or less; (3) routine long protocol of pituitary suppression followed by ovarian stimulation was used in all the previous stimulation cycles; (4) the rate of fragmented embryos was more than 50% in all the previous stimulation cycles using ejaculated sperm; (5) failed to conceive in all previous ejaculated stimulation cycles; (6) normal karyotype; (7) no microdeletions in Y chromosome were found in men with sperm concentration below 5 × 10^6^/ml. Exclusion criteria included: (1) azoospermia; (2) oocyte or sperm donation was involved.

**Figure 1:**
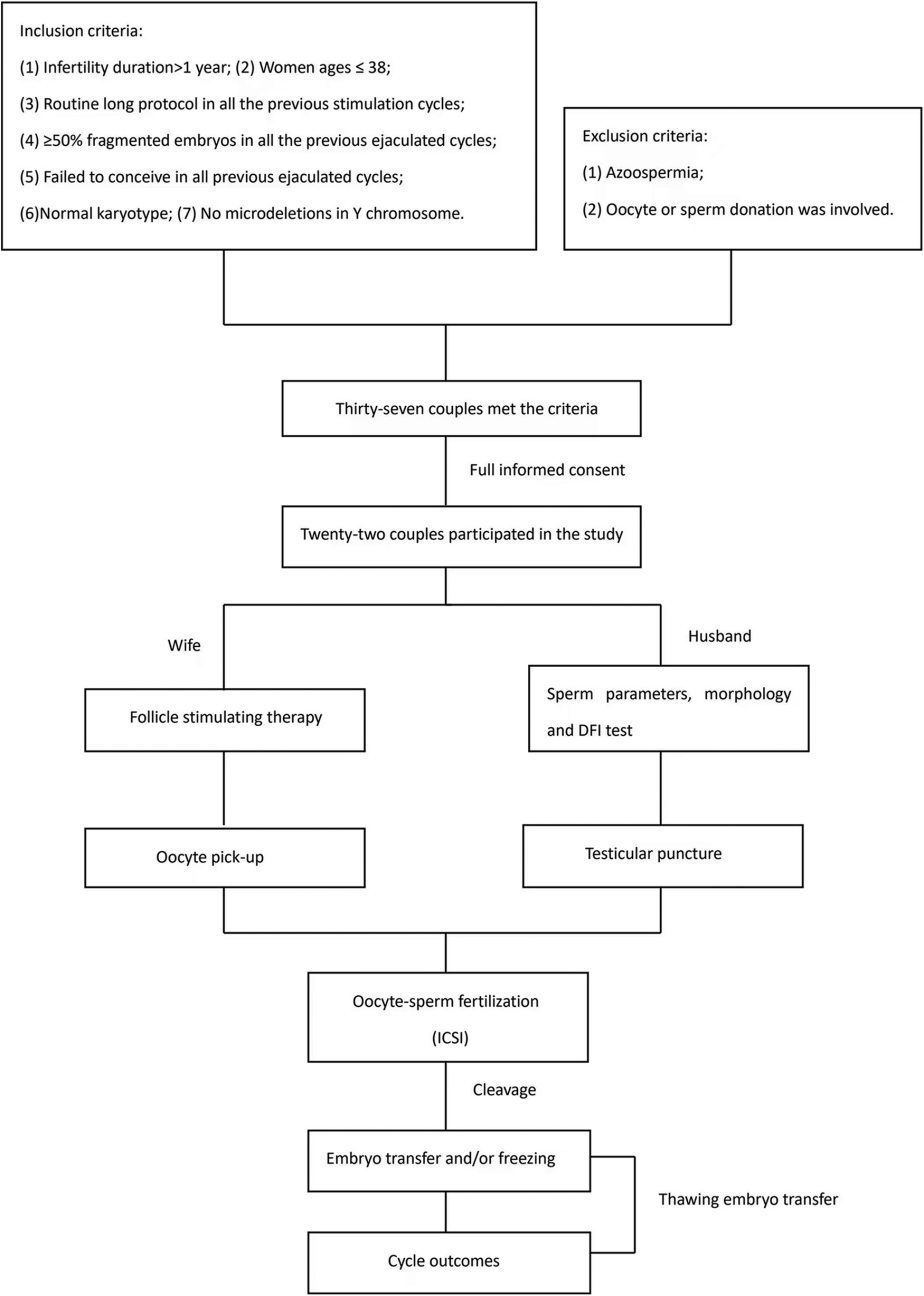
Selection criteria and the course of treatment for eligible patients.

Thirty-seven couples fulfilled the inclusion criteria, 22 of which agreed to participate in this study with full informed consent. The normal fertilization rate, transferable embryo rate, fragmented embryo rate and cycle outcomes were compared between EJA-sperm group and TESTI-sperm group. Figure 1 shows the course of treatment for eligible patients.

### Clinical examination and sperm analysis

All couples were offered determination of endocrine hormone. All women underwent ultrasound scans of their uterus and ovaries. Specimens were assessed for pH, volume, count, motility, and morphology according to the World Health Organization (WHO) manual 5 Standard Andrology criteria (19). Sperm concentration and motility were performed by the Sperm Class Analyser (SCA, version 5.0, MICROPTIC S.L, Barcelona, Spain) computer-assisted sperm analysis system (20).

Sperm DFI was assessed by the Sperm Chromatin Dispersion (SCD) test (21). Commercial kits (BRED LIFE SCIENCE, Shenzhen, China) were used and the main steps were as follows. Samples were diluted in mHTF medium, resulting in sperm concentrations between 5 × 10^6^/ml and 10 × 10^6^/ml. The suspensions were mixed with 1% low-melting-point aqueous agarose (to obtain a 0.7% final agarose concentration) at 37°C. Fifty µl of the mixture was transferred onto a slide pre-coated with 0.65% standard agarose dried at 80°C, covered with a coverslip and solidified at 4°C for 4 min. The coverslip was carefully removed, and the slide was immediately immersed horizontally in a tray with freshly prepared acid denaturation solution (0.08 N HCl) for 7 min at 22°C in the dark. The slide was then transferred to a tray with neutralizing and lysing solution 1 for 10 min at room temperature, followed by incubation in neutralizing and lysing solution 2 for 5 min. The slide was thoroughly washed, dehydrated and air dried. Cells were finally stained with Wright's dye for bright field microscopy. Sperm was judged to contain DNA fragments when the thickness of the unilateral halo was less than or equal to one-third of the minimum diameter of the sperm head. The DNA fragmentation rate was calculated by viewing 200 sperm under a light microscope at 400× magnification.

### Testicular sperm retrieval

**Figure 2:**
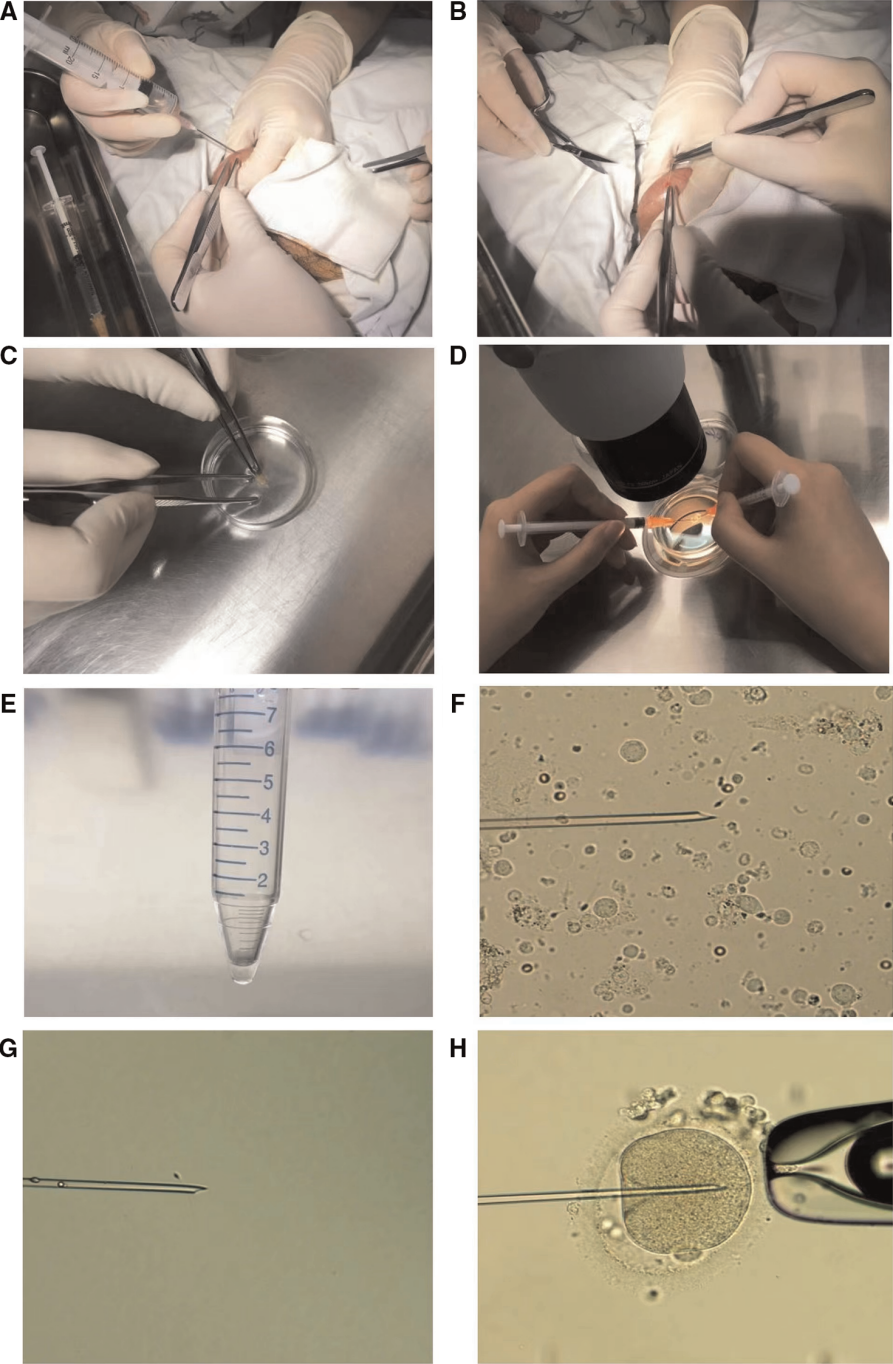
The procedures of testicular puncture, testicular sperm treatment and ICSI. (**A**) Testicular parenchyma was percutaneously aspirated using 20 ml syringes with 18 g needles. (**B**) The seminiferous tubules were pulled out by sterile tweezers and cut with scissors. (**C**) A mass of seminiferous tubules was transferred to a petri dish containing 3 ml of culture medium. (**D**) The seminiferous tubules were mechanically minced using the needled-tuberculin syringes. (**E**) About 20 uL of medium was left above the pellet after centrifugation. (**F**) A testicular sperm was selected and about to be sucked into the microneedle. (**G**) A testicular sperm was immobilized by the microneedle. (**H**) A testicular sperm was injected into a MII oocyte.

Retrievals were performed by testicular sperm aspiration (TESA) (22). All procedures were carried out under local anesthesia. Testicular parenchyma was percutaneously aspirated using 20 ml syringes with 18 g needles (Figure 2A). The seminiferous tubules were pulled out by sterile tweezers and cut with scissors (Figure 2B). The extracted testicular tissue was placed into a dish containing G-MOPS PLUS medium (Vitrolife Sweden AB., Västra Frölunda, Sweden) (Figure 2C) and blood clots were removed using the needled-tuberculin syringes. After initial washing, the seminiferous tubules were transferred to a new dish containing fresh G-MOPS PLUS and then were mechanically minced using the needled-tuberculin syringes (23) (Figure 2D). The cell suspensions were examined under the inverted microscope (Olympus Corp., Tokyo, Japan) to confirm the presence of motile sperm. Successful retrieval was defined as the presence of an adequate number of motile sperm for ICSI. Testicular sperm was used partly for ICSI and partly for SCD test. If the testicular tissue obtained by puncture was small, testicular spermatozoa would be used only for ICSI.

### Sperm specimen collection and processing

#### Ejaculated sperm

Semen samples were collected by masturbation after 2–7 days abstinence. If the patient's previous semen DFI was higher than 30%, part of the semen was used for SCD testing and the other part was subjected to gradient centrifugation. Firstly, the semen was processed by two-layer sperm separation medium Isolate® (Irvine Scientific Inc., Santa Ana, CA, United States) gradient centrifugation for 15 min. The pellet was then re-suspended in K-SIFM-100 medium (Cook Australia, Pty, Ltd., Queensland, Australia) and washed twice by centrifugation at 500 g for 5 min. The supernatant was carefully removed, leaving about 0.1–0.2 ml of medium above the pellet. Finally, the sperm suspension was kept at 37°C 6% CO2 for ICSI.

#### Testicular specimen

After mechanical mincing of the seminiferous tubules, cell suspensions were transferred to a centrifuge tube. It took about 5 min for the large tissues to settle down. The supernatant was then transferred to a fresh tube and centrifuged at 500 g for 10 min, leaving about 20 µl of medium above the pellet (Figure 2E). Finally, the resulting pellet was charged in a dish under the oil until used for ICSI. Only motile and morphologically normal or nearly normal sperm was chosen for injection (Figures 2F–H).

If the testicular tissue obtained by the puncture was about the size of a soybean and a certain amount of sperm was found under the microscope, the final testicular sperm suspensions were adjusted to about 50 µl. Five µl of the suspensions were added to the ICSI droplet and the remaining 45 µl suspensions were mixed with 1% low-melting-point aqueous agarose (to obtain a 0.7% final agarose concentration) at 37°C. The follow-up testing process was the same as the SCD method of semen spermatozoa.

### Ovarian stimulation and sperm-oocyte fertilization

Ovarian stimulation was performed using the routine long protocol of pituitary suppression followed by ovarian stimulation. We administered hCG 5,000 IU when at least 3 follicles were about 18 mm in diameter. Oocyte retrieval was performed by vaginal ultrasound-guided follicular puncture approximately 35–36 h after hCG administration. Forty hours after hCG administration, the oocytes were denuded of cumulus cells and the metaphase II (MII) oocytes were selected for injection (3). Oocytes were examined for fertilization 17–18 h after injection.

Figure 2 showed the whole process from testicular puncture to ICSI.

### Embryo scoring and embryo transfer

Evaluation for early embryonic cleavage was performed every 24 h after fertilization examination. Morphological evaluation of the embryos at cleavage stage was implemented according to the Istanbul consensus (18). In our center, high-quality embryos are the sum of grade A embryos and grade B embryos.

**Figure 3:**
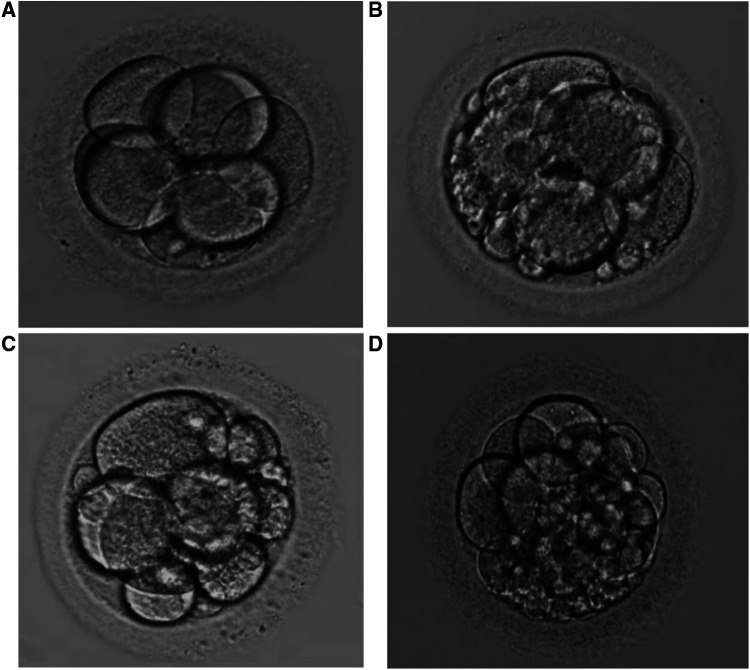
(**A–C**) represent embryos of grade (**A–C**), respectively. The fragment area in Figure 3. (**D**) is judged to be 1/3 of embryonic surface.

Fragmented embryo was defined as an embryo in which fragments account for more than one third of the embryonic surface area (seen in Figure 3).

Embryos of grade A, B or C were transferred or frozen on day 3 after oocyte retrieval. Fragmented embryos with four or more cells on day 3 were continuously cultured for 2 or 3 days and only those embryos forming blastocysts better than grade 3CC could be transferred or frozen (24). Fragmented embryos with fewer than four cells were directly discarded. Transferable embryos are the sum of embryos with grade A, B or C and blastocysts better than grade 3CC formed by fragmented embryos.

Fresh embryo transfers were usually performed on day 3. Fresh embryo transfer was canceled in patients with ovarian hyperstimulation syndrome (OHSS) predisposition or uneven endometrium.

The calculation methods of relevant parameters are as follows:

Normal fertilization rate% = 2PN oocytes /mature oocytes
Fragmented embryo rate% = Fragmented embryos/2PN embryos
Number of transferable embryos = Total number of embryos with grade A, B or C and blastocysts better than grade 3CC formed by fragmented embryos
Transferable embryo rate% = Transferable embryos/2PN embryos
Implantation rate% = gestational sacs/embryos transferred
All the above parameters are based on normal fertilization.

### Embryo vitrification and thawing

Cryotop device and commercial freezing solutions (Kitazato Corp., Tokyo, Japan) were used for embryos or blastocysts vitrification. Before vitrification, laser treatment was used to induce shrinkage of fully expanded blastocysts (25). Embryos or blastocysts were firstly equilibrated in equilibration solution for 5–10 min. Subsequently, embryos or blastocysts were put into vitrification medium and loaded onto the surface of the cryotop with a minimum volume within 1 min.

On the day of thawing embryo or blastocyst transfer, embryos or blastocysts were firstly transferred to 37°C thawing solution for 1 min, followed by 3 min in diluent solution and then washed twice in washing solution for 5 min. Warmed embryos or blastocysts were then cultured for at least 2 h prior to further evaluation. Cleavage-stage embryos with more than half the blastomeres surviving or re-expanded blastocysts were judged to be survived. Only survived embryos or blastocysts could be transferred.

### Pregnancy assessment and luteal support

Luteal support by 40 mg progesterone per day intramuscularly started 1 day after oocyte retrieval and continued until the establishment of pregnancy. Serum β-hCG assay was performed 15 days after oocyte retrieval. Clinical pregnancy was defined as a visible sac on the fifth gestational week.

### Statistical analysis

Descriptive parameters were expressed as mean ± SD. Differences in means between two variables were calculated using Student's *t*-test whereas *χ*^2^ and Fisher's exact tests were used for comparison of proportions. Calculations were performed by IBM SPSS 21.0 software (SPSS Inc., Chicago, IL, United States). *P*-values of less than 0.05 were considered statistically significant.

## Results

### Patient characteristics

Thirty-seven couples fulfilled the inclusion criteria, 22 of which agreed to participate in this study. The 22 couples were primary infertility with their husbands. The duration of infertility was from 2 to 7 years. Nineteen women had a normal body mass index (BMI), with 2 overweight patients and 1 underweight patient. The number of antral follicles during menstruation ranged from 3 to 10. Twenty-one women had no diseases that significantly affected pregnancy except one patient with polycystic ovary syndrome (PCOS). The process of ovarian stimulation was successful with no serious complications except one case of OHSS.

### Examination and treatment of the male spouse

Sperm morphological analysis was performed for 18 patients except 4 cryptozoospermic patients. Sperm DFI test was performed for 15 patients except 4 cryptozoospermic patients and 3 patients with sperm concentration below 1 × 10^6^/ml. There were 8 cases with asthenoteratozoospermia, 4 cases with cryptozoospermia, 4 cases with OTA, 2 cases with oligoasthenozoospermia, 2 cases with oligoteratozoospermia, 1 case with asthenozoospermia, and 1 case with teratozoospermia. Five patients whose sperm DFI was higher than 30% received oral antioxidant treatment for a minimum of 3 months before entering the stimulation cycle (26, 27). These 5 patients were reassessed for sperm DFI on the day of oocyte pick-up, showing that two patients’ sperm DFI had fallen into the normal range.

**Figure 4:**
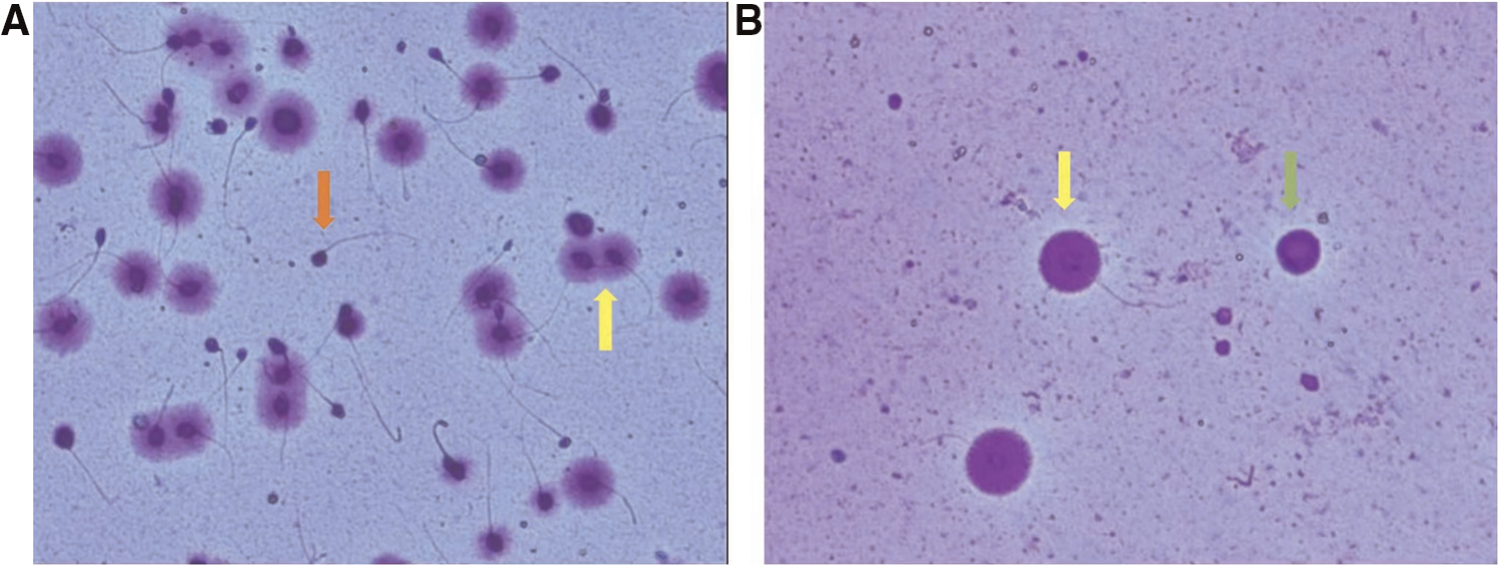
Image of sperm DNA fragment detected by SCD method ((**A**) semen sperm; (**B**) testicular sperm). Spermatozoa with no halo of chromatin dispersion represent those with fragmented DNA (red arrow). Spermatozoa with haloes of chromatin dispersion represent those with nonfragmented DNA (yellow arrow). The green arrow represents a spermatogenic cell. In contrast to mature sperm, spermatogenic cells have large, round nuclei and no tails.

No complications such as hemorrhage and infection occurred after testicular puncture. Sufficient testicular spermatozoa were obtained for both ICSI and SCD detection in 7 patients (seen in Figure 4). Testicular sperm DFI was significantly lower than that of ejaculated sperm (11.6% ± 4.9% vs. 21.2 ± 8.9%, *p* < 0.001). Sperm parameters, sperm DFI (in 5 patients with previous high sperm DFI, the reassessed results were shown), testicular sperm DFI and male endocrine hormone level are shown in Table 1.

**Table 1:** Sperm DFI, semen parameters and hormone levels of male partners.

	Asthenoterato- zoospermia	Crypto- zoospermia	OTA	Oligoastheno- zoospermia	Oligoterato- zoospermia	Astheno- zoospermia	Terato- zoospermia
No.	8	4	4	2	2	1	1
Volume (ml)	2.5 ± 9.3	2.7 ± 0.4	2.3 ± 1.5	2.1 ± 0.6	4.5 ± 0.7	3.0	4.0
Concentration (×10^6^/ml)	48.2 ± 28.8		7.2 ± 5.3	10.2 ± 3.7	0.5 ± 0.4	38.7	40.6
PR (%)	6.9 ± 6.6		8.0 ± 8.4	15.3 ± 11.6	43.8 ± 8.8	23.1	45.5
Morphology (%)	1.4 ± 1.0		1.3 ± 0.9	4.3 ± 0.4	0.5 ± 0	4.5	2.0
S-DFI (%)	21.1 ± 9.8		27.7 ± 7.8	12.8 ± 1.1	25.5	15.5	
(Mean of sum)	21.2 ± 8.9						
T-DFI (%)	14.3 ± 8.1	12.5	10.5		9.0	15.5	5.5
(Mean of sum)	11.6 ± 4.9^a^						
FSH (iu/L)	6.65 ± 2.39	9.85 ± 5.43	5.35 ± 2.69	8.45 ± 0.49	5.67 ± 3.58	5.23	4.10
LH (iu/L)	5.88 ± 2.54	6.0 ± 4.60	5.08 ± 2.20	5.51 ± 2.20	5.13 ± 3.35	3.45	5.21
T (µg/L)	2.93 ± 1.04	2.70 ± 0.65	3.81 ± 1.02	3.16 ± 0.50	3.19 ± 0.44	3.44	3.40

Patients with cryptozoospermia were not tested for majority of the sperm parameters.

Patients with sperm concentration below 1 × 10^6^/ml were not tested for sperm DFI.

S-DFI means DFI of semen sperm.

T-DFI means DFI of testicular sperm.

^a^ Testicular sperm DFI was significantly lower than that of ejaculated sperm (*p* < 0.001).

### Ovulation induction, embryo quality and pregnancy

Twenty-two couples underwent 32 ejaculated and 23 testicular stimulation cycles in our hospital. All cycles were ICSI cycles. There was no significant difference in E2 and P levels on the day of HCG injection between EJA-sperm group and TESTI-sperm group. There was also no significant difference in age, normal fertilization rate and high-quality embryo rate between the two groups. The transferable embryo rate in TESTI-sperm group was significantly higher than that in EJA-sperm group (36.9% vs. 22.0%, *p* < 0.01). The fragmented embryo rate in TESTI-sperm group was significantly lower than that in EJA-sperm group (61.2% vs. 75.7%, *p* < 0.05) (Seen in Table 2).

**Table 2:** Embryonic and pregnancy outcomes after ICSI of ejaculated versus testicular spermatozoa cycles.

	EJA-sperm group	TESTI-sperm group	*p*- value
Stimulation cycles, *n*	32	23	
Cancelled transfer cycles, *n*	8	4	
Retrieved oocytes, *n*	292	178	
Metaphase II oocytes, *n*	249	153	
Transferable embryos, *n*	38	38	
Female age, *y*	30.5 ± 4.1	31.5 ± 4.0	0.879
Male age, *y*	34.3 ± 7.2	35.1 ± 7.1	0.930
E2 on day of hCG injection, pg/ml	3209.8 ± 1377.0	2778.1 ± 1390.4	0.614
*p* on day of hCG injection, ng/ml	3.44 ± 3.03	2.16 ± 2.13	0.053
Normal fertilization rate (%)	70.3 (175/249)	68.0 (104/153)	0.656
High-quality embryo rate (%)	0.6 (1/173)	3.9 (4/103)	0.066
Transferable embryo rate (%)	22.0 (38/173)	36.9 (38/103)	0.008^a^
Fragmented embryo rate (%)	75.7 (131/173)	61.2 (63/103)	0.014^b^
Implantation rate (%)	0 (0/38)	34.2 (13/38)	0.000^c^

^a^ The transferable embryo rate in TESTI-sperm group was significantly higher than that in EJA-sperm group (36.9% vs. 22.0%, *p* < 0.01).

^b^ The fragmented embryo rate in TESTI-sperm group was significantly lower than that in EJA-sperm group (61.2% vs. 75.7%, *p* < 0.05).

^c^ The implantation rate in TESTI-sperm group was significantly higher than that in EJA-sperm group (34.2% vs. 0%, *p* < 0.001).

**Figure 5:**
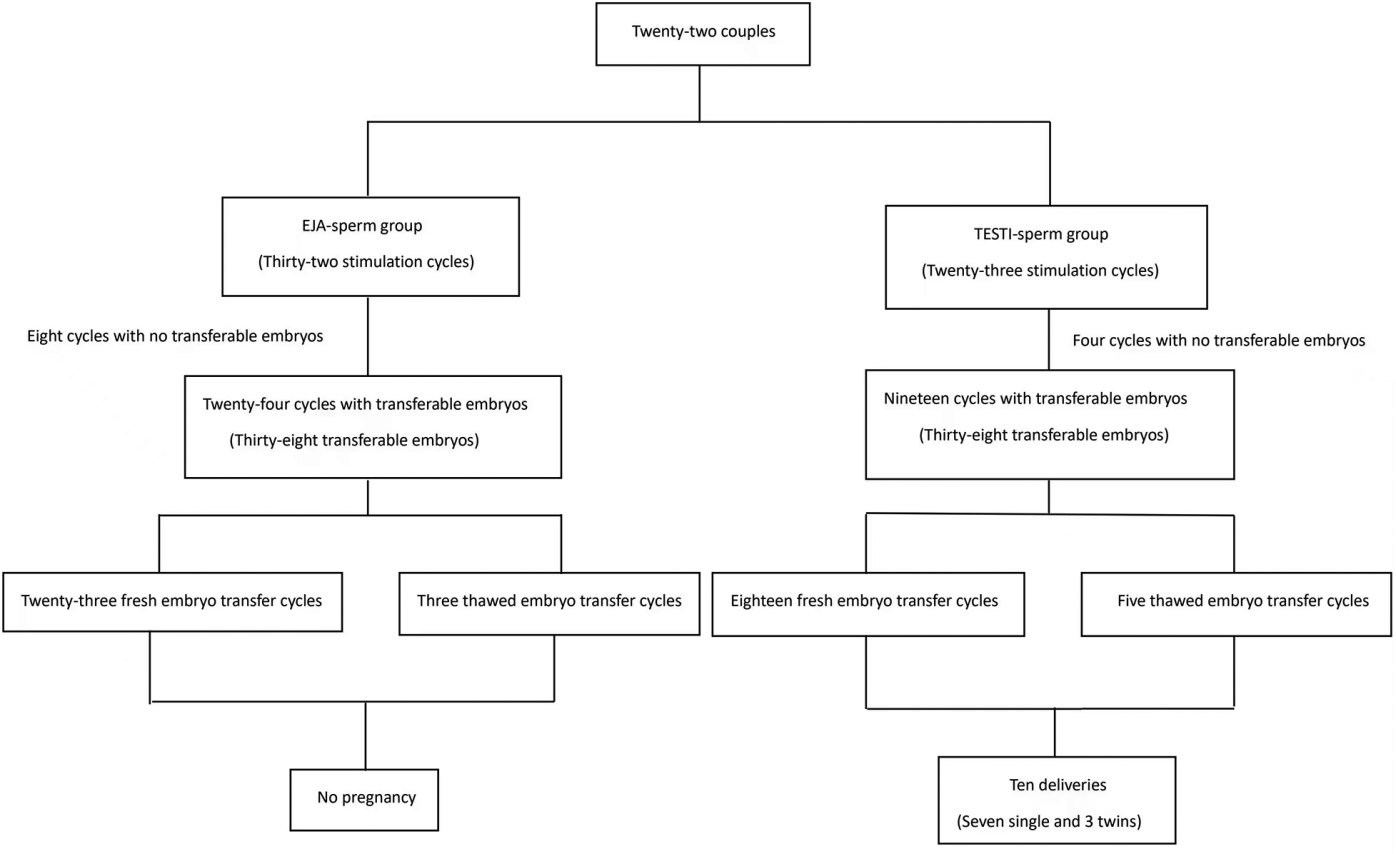
Information on ovulation induction, embryo transfer and cycle outcomes in EJA-sperm group and TESTI-sperm group.

Embryo transfers were cancelled in 8 ejaculated and 4 testicular stimulation cycles because of no transferable embryos. Fresh embryo transfer was canceled in one patient with OHSS predisposition. Thirty-eight transferable embryos were obtained from 24 ejaculated stimulation cycles, but no pregnancy was achieved after 23 fresh embryo transfer cycles and 3 thawed embryo transfer cycles. Thirty-eight transferable embryos were obtained from 19 testicular stimulation cycles. Ten couples achieved successful deliveries including 7 single and 3 twins after 18 fresh embryo transfer cycles and 5 thawed embryo transfer cycles, with an implantation rate of 34.2% which was significantly higher than that of EJA-sperm group (*p* < 0.001). The data is shown in Table 2. Information on ovulation induction, embryo transfer and cycle outcomes in EJA-sperm group and TESTI-sperm group is shown in Figure 5.

## Discussion

Surgically obtained testicular sperm for ICSI has been widely used in patients with ejaculatory dysfunction or azoospermia who have failed to respond to medical or surgical treatment. Although controversy exists, there is increasing evidence that using testicular sperm can achieve satisfactory cycle outcomes. In this study, we found that patients with large and scattered embryo fragments can also try using testicular sperm, and the results were encouraging.

In a well-run reproductive center, the environment in which embryos are grown and the laboratory operations of the embryologists are precisely controlled. In this context, embryo quality depends more on the quality of the gametes. Several studies have confirmed that testicular sperm have lower DNA damage compared with ejaculated sperm in patients with abnormal ejaculated sperm DNA integrity (5, 9, 28). Hammoud et al. (2017) suggested that testicular sperm DNA appears to be significantly less damaged than epididymal sperm DNA, and so testicular sperm should be used in preference for ICSI to treat male patients with obstructive azoospermia (29). These observations confirm the working hypothesis that most of the DNA damage observed in ejaculated sperm result from alterations occurring at the post-testicular level. Male patients usually abstain from sex for 3–7 days before oocyte retrieval, which tends to increase the DNA fragmentation in ejaculated sperm (6, 7). Sperm preparation processes including gradient centrifugation and washing can also increase the sperm DNA fragmentation (8, 9). Karimi et al. (2020) found that sperm DNA fragmentation increased significantly with the extension of culture time (30). On the basis of the above analysis, we believe that testicular sperm bypasses genital tract, long abstinence, and gradient centrifugation process and thus is speculated of having better quality compared with ejaculated or epididymal sperm.

To minimize confounding variables, we selected the same patients who used both semen and testicular spermatozoa, and all patients received the same ovulation induction protocol. The patients used semen sperm first and testicular sperm later. Therefore, age may be a confounding factor although there was no significant difference in age between the two groups. In this study, some couples received fresh embryo transfer while others had frozen-thawed embryo transfer. Frozen-thawed embryo transfer is suggested to have inherent benefits and better cycle outcomes than fresh embryo transfer (31). This could also generate bias in the results.

In this study, we obtained a certain amount of testicular tissue from 7 patients for testicular sperm DFI detection, and the results further showed that testicular sperm DFI was significantly lower than that of semen sperm. Meanwhile, our study verified that testicular sperm significantly reduced embryo fragmentation and improved cycle outcome, which may further confirm the application value of testicular sperm and then broaden the use of testicular sperm.

Five patients received oral antioxidant therapy for at least 3 months, and only 3 patients showed that sperm DFI was still higher than 30% after reassessment, suggesting that high DFI was not the only reason for the high rate of embryo fragmentation. All the patients in this study had abnormal sperm parameters, which may also be responsible for high embryo fragmentation. Especially for patients with severe oligozoospermia, OTA, or cryptozoospermia, the probability of abnormal sperm being selected is increased, leading to an increased probability of poor-quality embryo formation. On the other hand, the rate of fragmented embryos in testicular group was still much higher than that in the general population. Did female factors participate? Or can’t the testicular sperm of these 22 men compete with the ejaculated sperm from normal, fertile men? Perhaps having an egg or sperm donation would help, but these couples refused to use this method. Therefore, it can be concluded that the involvement of male factors in the formation of highly fragmented embryos is certain, but the presence of female factors is not clear.

Strict case screening conditions and couples' reluctance to undergo testicular puncture are the main reasons for the small sample size of this study. Before starting the study, couples were informed of the small risks of testicular puncture, such as hematoma and infection. Twenty-two men agreed to undergo the invasive surgery. In all cases of testicular puncture, sufficient motile sperm were found for injection. No complication occurred in all cases, indicating that testicular puncture is safe.

## Conclusions

Testicular puncture is simple and safe with little injury. Testicular sperm DFI was lower than that of ejaculated sperm. Our study found that testicular sperm could reduce embryo fragmentation and improve cycle outcome in couples with previous ART failure because of high rate of fragmented embryos after using ejaculated sperm. So, using testicular sperm is an alternative for such couples. To some extent, our single-center non-randomized controlled study with small sample size affects the credibility of our conclusions. Large samples, multicenter studies or RCT are needed to further confirm the application value of testicular sperm.

## Data Availability

The raw data supporting the conclusions of this article will be made available by the authors, without undue reservation.
